# Validity and reliability of criterion based clinical audit to assess obstetrical quality of care in West Africa

**DOI:** 10.1186/1471-2393-12-118

**Published:** 2012-10-29

**Authors:** Catherine M Pirkle, Alexandre Dumont, Mamadou Traore, Maria-Victoria Zunzunegui

**Affiliations:** 1 Department of Social and Preventive Medicine, Université de Montréal, Montréal, Canada; 2 Research Institute for Development, Université Paris Descartes, Sorbonne Paris Cité, UMR 216, Paris, France; 3 URFOSAME, District health centre of the Commune V, Bamako, Mali; 4 Hospital Research Centres of the University of Montreal Hospital Complex (CRCHUM), Montréal, Canada

**Keywords:** Criterion-based clinical audit, Questionnaire development, Quality of care, Validity, Reliability, Resource-limited settings

## Abstract

**Background:**

In Mali and Senegal, over 1% of women die giving birth in hospital. At some hospitals, over a third of infants are stillborn. Many deaths are due to substandard medical practices. Criterion-based clinical audits (CBCA) are increasingly used to measure and improve obstetrical care in resource-limited settings, but their measurement properties have not been formally evaluated. In 2011, we published a systematic review of obstetrical CBCA highlighting insufficient considerations of validity and reliability. The objective of this study is to develop an obstetrical CBCA adapted to the West African context and assess its reliability and validity. This work was conducted as a sub-study within a cluster randomized trial known as QUARITE.

**Methods:**

Criteria were selected based on extensive literature review and expert opinion. Early 2010, two auditors applied the CBCA to identical samples at 8 sites in Mali and Senegal (n = 185) to evaluate inter-rater reliability. In 2010–11, we conducted CBCA at 32 hospitals to assess construct validity (n = 633 patients). We correlated hospital characteristics (resource availability, facility perinatal and maternal mortality) with mean hospital CBCA scores. We used generalized estimating equations to assess whether patient CBCA scores were associated with perinatal mortality.

**Results:**

Results demonstrate substantial (ICC = 0.67, 95% CI 0.54; 0.76) to elevated inter-rater reliability (ICC = 0.84, 95% CI 0.77; 0.89) in Senegal and Mali, respectively. Resource availability positively correlated with mean hospital CBCA scores and maternal and perinatal mortality were inversely correlated with hospital CBCA scores. Poor CBCA scores, adjusted for hospital and patient characteristics, were significantly associated with perinatal mortality (OR 1.84, 95% CI 1.01-3.34).

**Conclusion:**

Our CBCA has substantial inter-rater reliability and there is compelling evidence of its validity as the tool performs according to theory.

**Trial registration:**

Current Controlled Trials ISRCTN46950658

## Background

Worldwide, approximately 1000 women die each day during pregnancy or of childbirth-related causes. More than half of these occur in sub-Saharan Africa [1]. Further, an estimated three million babies are stillborn every year; stillbirths account for the majority of perinatal deaths in developing countries [2]. One in three stillbirths occur during delivery and in most cases, could have been prevented with improved intrapartum management of the mother [2]. Stillbirth and maternal mortality are strongly correlated; both decline with improved access to caesarean section and skilled attendance [3]. Experts agree that one way to reduce maternal and perinatal mortality is to encourage women to routinely deliver in health facilities [1,4,5]. For such a strategy to work, women must have confidence in the health system [6] and the system itself should not contribute to mortality.

In Mali and Senegal, over one percent of women die giving birth in referral hospitals [7]. National rates of perinatal mortality are estimated at 50 per 1000 births [2], but levels at referral hospitals may be much greater. Such high case fatality suggests poor medical practice. Gross deficiencies in the quality of care provided to women during childbirth are widely recognized in West Africa [8]. Insufficiencies in health personnel training, shortages of basic obstetrical equipment, and dramatic, even violent interactions between care givers and parturients have been reported [8,9]. Limited quantitative work supports these results; up to 70 percent of in-hospital maternal deaths may be avoidable with improved quality of care [10]. Nonetheless, maternal mortality is still a rare event and estimators are often unstable [11]. This makes it difficult to directly link maternal mortality to quality of care. Perinatal mortality, however, is more common and frequently directly related to the care episode [12].

At present, we have little indication of the amplitude of the quality problem or which aspects of medical practice to target. One of the reasons for this knowledge gap is the difficulty inherent to measuring the quality of obstetrical practice at the patient level. There are several methods for measuring medical practice: standardized patients, direct observation, vignettes, and chart abstraction. Standardized patients require a trained actor to engage medical professionals in a clinical examination related to a topic of research interest. For obvious reasons, it is impossible for an actor to convincingly recreate the birth process. It is also possible to measure quality of care through direct observation, but this method is subjective and risks the Hawthorne effect. Finally, there are vignettes and chart abstraction. Vignettes are written scenarios involving a fictitious patient [13]. Work in high-income countries show that vignettes have promising measurement properties [14], but there are limitations: they measure individual provider knowledge versus team practice, no validation studies have been conducted in low-income countries, and current tools target physicians versus midwives or other lower-cadre staff.

Chart abstraction is a general term for when researchers retrieve predefined information from patient medical records and compare that information against agreed-upon standards of care. A criterion-based clinical audit (CBCA) is a specific type of chart abstraction that can be effectuated by non-medically qualified audit assistants. Assistants screen the medical records of patients and extract relevant data. Standardized criteria for evaluating good quality care are predetermined and then compared against extracted data to evaluate whether or not a minimal standard of care has been met [5,14]. CBCA are gaining traction in the domain of obstetrical care in resource-limited settings, as they can dually serve to measure and improve care [5,14-22].

In 2011, we published a systematic review of obstetrical CBCA in resource-limited settings [15]. The review highlighted insufficient considerations of CBCA reliability and validity. This article has two objectives. The first objective was to report on the development a CBCA instrument for West Africa. The second objective was to address the gaps identified through the systematic review, namely to evaluate inter-rater reliability and construct validity. To assess construct validity, e.g. whether the instrument is performing according to theory, we followed Donabedian’s conceptualization of quality of care: 1) Given an adequate setting and equipment (structure), quality medical care (process) will follow and 2) In the absence of proper medical procedures (process), good health outcomes will not be achievable [16]. If our instrument is valid, we hypothesize that we will observe the following: 1) A positive correlation between structure and process measures of quality of care 2) A negative correlation between process measures of quality of care and adverse maternal and perinatal outcomes.

## Methods

### Background

This is a sub-study within a cluster randomized trial called QUARITE [7]. The trial assessed a multifaceted quality improvement intervention known as the ALARM International Program [7]. The aim of the ALARM intervention is to reduce facility maternal mortality. QUARITE began in September 2007 and was completed in December 2012. It took place in 46 out of 49 eligible referral hospitals in Mali and Senegal. Referral hospitals in both countries treat complicated deliveries and receive evacuations from lower order health centres. Hospitals were considered eligible for study if their maternity registered a minimum of 800 births per year and could provide comprehensive emergency obstetrical care [17]. The primary endpoint measure of QUARITE is overall facility-based maternal mortality (number of maternal deaths divided by the number of women giving birth in the facility). The trial also measured (described in subsequent sections): (i) resource availability; (ii) intrapartum quality of care; and (iii) and perinatal mortality [7].

### Description of the CBCA questionnaire

From 2008 to 2010, we developed a CBCA adapted to the West African context to measure *intrapartum quality of care*. We focus on quality of care during labour, delivery, and the first 24 hours postpartum because most maternal deaths occur during this period [4] and because the duration of hospital stay for normal deliveries is usually less than 24 hours after birth in the region. The CBCA is organized in five domains: history taking, clinical examination, laboratory analyses, monitoring during birth, and postpartum follow-up. The organization of the audit reflects the basic steps expected of a medical team following a woman through delivery and birth.

### Generation of the item pool

Criteria were generated by reviewing the literature (peer reviewed articles, Cochrane reviews, and the WHO reproductive health library), best-practice guidelines from the Royal College of Obstetricians and Gynaecologists, and expert opinion. Criteria selection was consistent with WHO methods for conducting an obstetrical CBCA [18]. Once a comprehensive item pool was compiled, C. Pirkle and A. Dumont removed criteria impossible to verify in a West African setting. C. Pirkle is a public health researcher with extensive experience in West African maternities and A. Dumont, an obstetrician/gynaecologist with 15 years of clinical practice and research experience in Senegal.

### Review of initial criteria pool with experts

Using the criteria selected in the previous step, a draft CBCA instrument was constructed and circulated to two Canadian and three West African obstetrician/gynaecologists. We asked the reviewers to evaluate the criteria included (or excluded) for relevance and clarity. The CBCA instrument was refined based on their suggestions and modifications (inclusions, exclusions, clarifications). All experts agreed on the final version of the CBCA instrument used during data collection. See Table 1 for the final criteria list.

**Table 1:** Final criteria (n=26) for the obstetrical CBCA questionnaire

**Domain**	**Criteria**
History taking	· Condition of the mother at arrival
	· Number of prenatal visits
	· Age
	· Gravidity
	· Parity
Clinical examination	· Uterine height
	· Cardiac frequency
	· Blood Pressure
	· Temperature
	· Foetal presentation
	· Foetal heart beat
	· Membranes/amniotic fluid
	· Cervical dilation
Laboratory analyses	· Blood type
	· Rhesus factor
	· HIV test
	· Syphilis test
Monitoring during birth	· Name of birth attendant
	· Qualification of birth attendant
	· Time of placental expulsion
	· Oxytocin given
	· Time of birth given
Postpartum monitoring	· Follow-up examination
	· Exit examination
	· Date of discharge
	· Vital status of the infant at birth

### CBCA questionnaire format and scoring

In a typical CBCA, auditors review patients’ medical records to ascertain whether pre-specified criteria have been met [18,19]. A CBCA is a checklist of pre-specified standards of good quality care. For example, the auditor may “check-off” if a patient’s blood pressure, cardiac frequency, and temperature were taken. For each affirmative response related to an expected standard care, a point is given. Our questionnaire contained 26 criteria. The CBCA questionnaire was scored according to the percentage of criteria attained. Thus, if 20 criteria were attained during the audit of a given medical record, then the score for that record would be 20/26 or 76.9%.

### Language

The CBCA questionnaire and all supporting documents (e.g. the procedures manual and lexicon of common medical abbreviations) were written in French. An English translation of the final CBCA questionnaire can be found in the Additional file 1 (the French version can be obtained upon request from CP). The English CBCA questionnaire was translated by the first author (CP) who is a native English-speaker. The translation was verified by the second author (AD), a French-speaking obstetrician-gynaecologist. Both CP and AD were involved in all phases of CBCA development.

### Data entry form and validity checks

An electronic version of the CBCA questionnaire was installed onto notebook computers with long battery lives. Validity checks were programmed to limit missing data and improve accuracy by detecting impossible responses (e.g. 77 versus 17 for maternal age). Auditors entered data directly into the electronic questionnaire. Data were exported to Microsoft Excel and other statistical software.

### Data collection and sampling strategy

**Figure 1:**
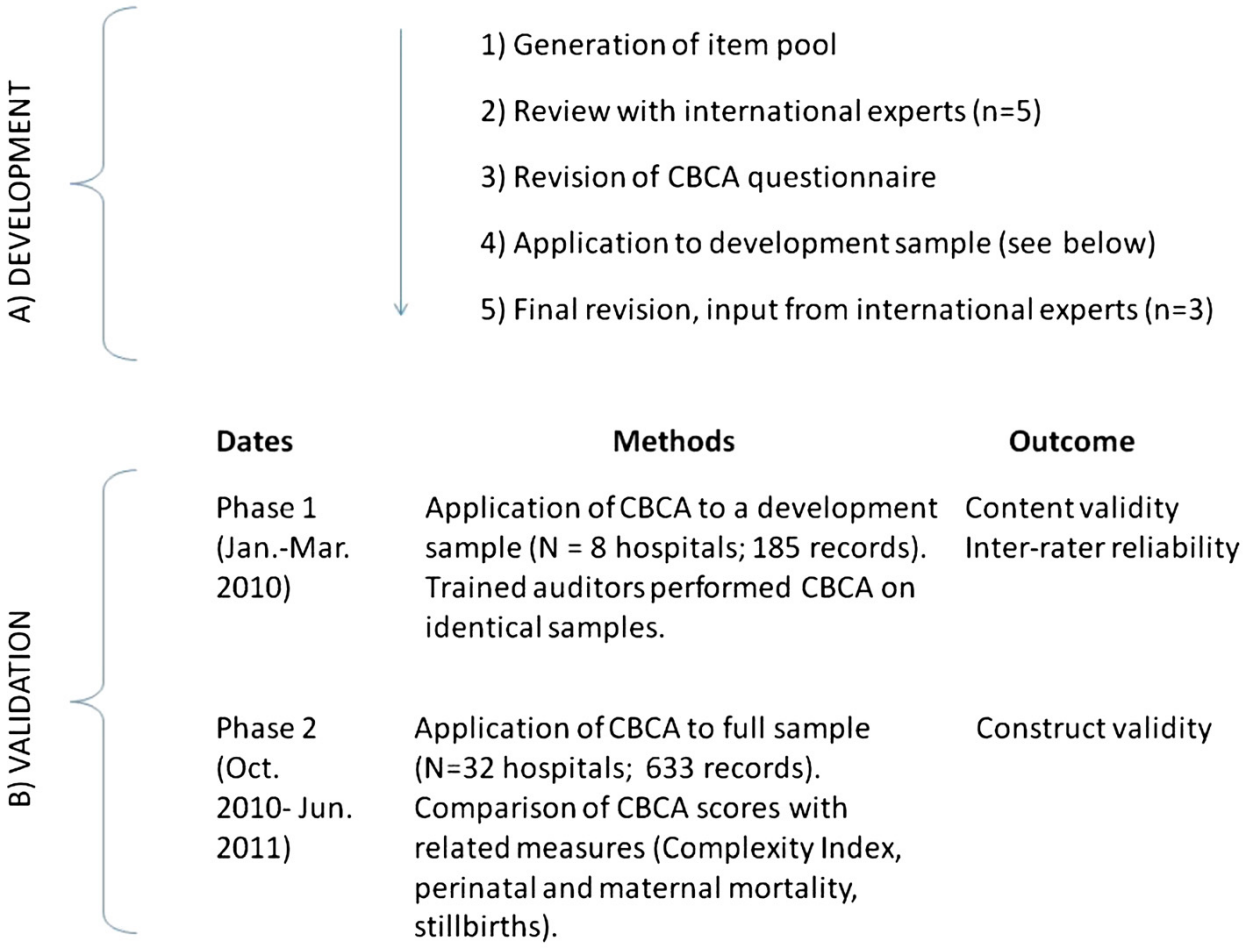
Schema depicting CBCA development and validation.

Figure 1 shows the different steps and dates in the CBCA development and validation. There were two phases of data collection. The first phase took place at 4 hospitals in Bamako, Mali and 4 hospitals in Dakar, Senegal. In this phase, we used a development sample of patient records to evaluate inter-rater reliability between two auditors and improve content validity. The second phase of data collection used a finalized version of the questionnaire and took place at 32 hospitals in Mali and Senegal (13 in Mali and 19 in Senegal). For logistic and financial reasons we were not able to include all 46 eligible QUARITE sites. Of the 32 included hospitals, 11 were located in the country capitals, 11 were regional and 10 were district hospitals. Data from the second phase of data collection were used to assess construct validity.

During the first phase of data collection, we audited 185 obstetrical records. Cases were identified from delivery-room birth registries and corresponding obstetrical records were sought. Most medical records were stored in the delivery room or the examination room of the head midwife. We audited, in reverse consecutive order, the most recent 20–25 deliveries having occurred at a given site. When a case’s medical record could not be found, the next most recent birth was selected. During the second phase of data collection, we followed essentially the same sampling strategy. In both countries, we used the delivery-room birth registry to locate and audit the last 15–20 women who gave birth starting with September 30, 2010. As during the first phase of data collection, when we could not find a woman’s record, the next consecutive record was retrieved. We corresponded the second phase of data collection with the national QUARITE coordinators’ trimestral visit of the hospital sites. During the coordinator’s visit, he/she verified data recorded on trial data collection sheets with information retrievable in various hospital registries and patient obstetrical records. This allowed us to triangulate multiple patient registries to maximize the number of retrievable patient records. We audited 661 medical records, of which 633 had complete data to calculate CBCA scores.

### Patient inclusion criteria

Because we are measuring intrapartum obstetrical care, we included the obstetrical records of all women admitted to hospital in labour with a foetus of at least 500 gms. Thus, women admitted for elective caesarean section were not included.

### Measures

#### Facility maternal and perinatal mortality

We calculated hospital maternal and perinatal mortality for the three month period around when the audits were conducted (July 1-September 30, 2011 in Senegal and September 1- November 30, 2011 in Mali). Maternal and perinatal mortality was calculated based on information recorded on a data sheet collected for every patient giving birth at QUARITE hospitals (see patient demographic and obstetric variables below). These dates correspond to the national coordinators’ trimestral visit to the hospitals in each country and provide a large enough window to calculate reliable estimates of facility maternal and perinatal mortality. Facility perinatal mortality is defined as the number of newborn deaths (including stillbirths) occurring prior to the woman’s discharge divided by the number of livebirths occurring at the hospital during the eligible three-month study window. Here, facility maternal mortality is defined as the number of maternal deaths occurring at a given hospital during the study period divided by the total number of livebirths during that same period. Both ratios are given per 1000 livebirths.

#### Indicator of facility resource availability

The QUARITE trial annually collects data on hospital material and human resources. The Complexity Index we employed is derived from the WHO Global Survey on Maternal and Perinatal Health [20]. It is comprised of eight categories describing: 1) basic services, 2) screening tests, 3) basic emergency obstetrical resources, 4) intrapartum care, 5) general medical services, 6) anaesthesiology resources, 7) human resources and, 8) academic resources and clinical protocols. We used an Africa-specific grading scheme for the Index [21]. A list of services under each of the categories described above is classified as essential, comprehensive, or advanced. Each service classified as essential receives one point, each comprehensive service receives two points, and each advanced service gets 3 points. Points for the Complexity Index were summed up for each hospital. Scores vary from 0 to 100.

#### Patient demographic and obstetrical variables

As part of the larger QUARITE trial, a data sheet was completed for every woman who gave birth in participating hospitals; it includes the mother’s survival outcome, age, parity, number of prenatal visits, and previously diagnosed maternal conditions. The vital status of the infant at birth and at discharge was recorded in the QUARITE trial data sheet and verified by the auditor during the CBCA audit. For each patient, we merged audited data with that collected by the QUARITE trial using a unique patient identifier. During the CBCA audit, the data collector did not find the vital status of 23 infants at birth. According to the QUARITE database, all 23 infants were alive at discharge, but four had very low birth weights (less than 1500 gms). Given uncertainty between databases and the very low probability of the infants’ survival, we treated these four infants as perinatal deaths (though we conducted additional analyses treating the infants as alive).

### Statistical analyses- reliability and validity

Analyses were conducted in STATA 11 and SPSS 17. We stratified most analyses by country because assessments of reliability and validity are context specific [22] and because of differences between Malian and Senegalese health systems in terms of user fees and hospital decentralization. We calculated the mean, standard deviation, minimum and maximum CBCA scores. Using data from the first phase of data collection, we calculated the intra-class correlation coefficient to determine inter-rater reliability.

To assess construct validity, we followed Donabedian’s conceptualization of quality of care according to structure, process, and outcome (see introduction) [16]. The CBCA questionnaire measures the quality of obstetrical care at the process level (e.g. medical procedures and practice) while the Complexity Index measures structure. We first evaluated hospital-level associations. We expected a positive correlation between the Complexity Index and CBCA questionnaire. To look at correlations between CBCA scores and the Complexity index, we aggregated patient CBCA scores to calculate a mean quality of care score for each hospital (hospital CBCA scores). We used scatterplot graphs to visually assess the relationship between Complexity Index and hospital CBCA scores. Spearman’s rho, with one-tailed tests of significance, was used to evaluate the correlation between the two scores.

We also looked at the correlation between hospital CBCA scores and hospital rates of maternal and perinatal mortality. We expected a negative correlation between the CBCA questionnaire and adverse maternal and perinatal outcomes. We did this analysis in the same manner as we did for the Complexity Index and CBCA scores.

Finally, we looked at patient-level correlations. It is possible that a hospital may provide an overall high level of quality of care but that individual episodes of poor quality care could be correlated with adverse patient outcomes (stillbirth and early neonatal mortality). We selected a score of 70% to represent good quality of care; this was the upper quartile of scores for women treated in Senegal. For each section of the questionnaire, we calculated the percentage of stillbirths and early neonatal mortality in women with CBCA scores above and below 70%. For the full questionnaire, we also conducted sensitivity analyses looking at different thresholds of good quality care (scores of 60, 70, and 80). We used generalized estimating equations with an exchangeable matrix to evaluate the association between CBCA score and stillbirth and early neonatal mortality. We adjusted for country, Complexity Index score, and capital versus regional facility location. We also assessed potential confounding by patient characteristics including maternal age, parity, number of prenatal visits, and previously diagnosed maternal condition. If the coefficient for CBCA score changed by 10% or more, the variable was considered a confounder.

### Sample size

Estimates are given for an alpha of 0.05 and a beta of 0.20. For the estimation of inter-rater reliability, 40 medical charts per country were sufficient to estimate an intraclass correlation coefficient of 0.80, given a minimum acceptable level of reliability of 0.60 [23]. For the hospital level correlations (n=32), we calculated study power with the statistical package pwr of R. Based on a null hypothesis of a correlation coefficient of 0 against an alternative of 0.50, our power to detect a significant result is 0.84.

## Results

Mean patient CBCA scores differed significantly by country (p=0.000). In Senegal, mean patient criterion attainment was 60.0% (SD 11.4). In Mali, mean attainment was 73.4% (SD 12.0). Hospital complexity index scores varied from 50.0-89.0. Significantly more patients in Senegal were treated at hospitals with higher mean complexity scores than patients in Mali (72.7 vs 63.1%). Average hospital perinatal mortality was 135/1000 in Mali (range 25-270/1000) and 168/1000 in Senegal (range 13-390/1000). For maternal mortality, these numbers were 13/1000 (range 0-30/1000) in Mali and 12 (0-30/1000) in Senegal.

### Inter-rater reliability

According to the classification scheme proposed by Landis and Koch (1977), inter-rater reliability for the CBCA questionnaire was substantial to high [24]. Using data from the first phase of data collection, we sampled 96 obstetrical records in Senegal and obtained an ICC of 0.66 (95% CI 0.54-0.76). We sampled 89 obstetrical records in Mali and obtained an ICC of 0.84 (95% CI 0.77-0.89). Note that the upper limit of the confidence interval in Senegal is below the lower limit of the interval in Mali.

### Construct validity

#### Correlation between hospital CBCA scores and hospital complexity index score

**Figure 2:**
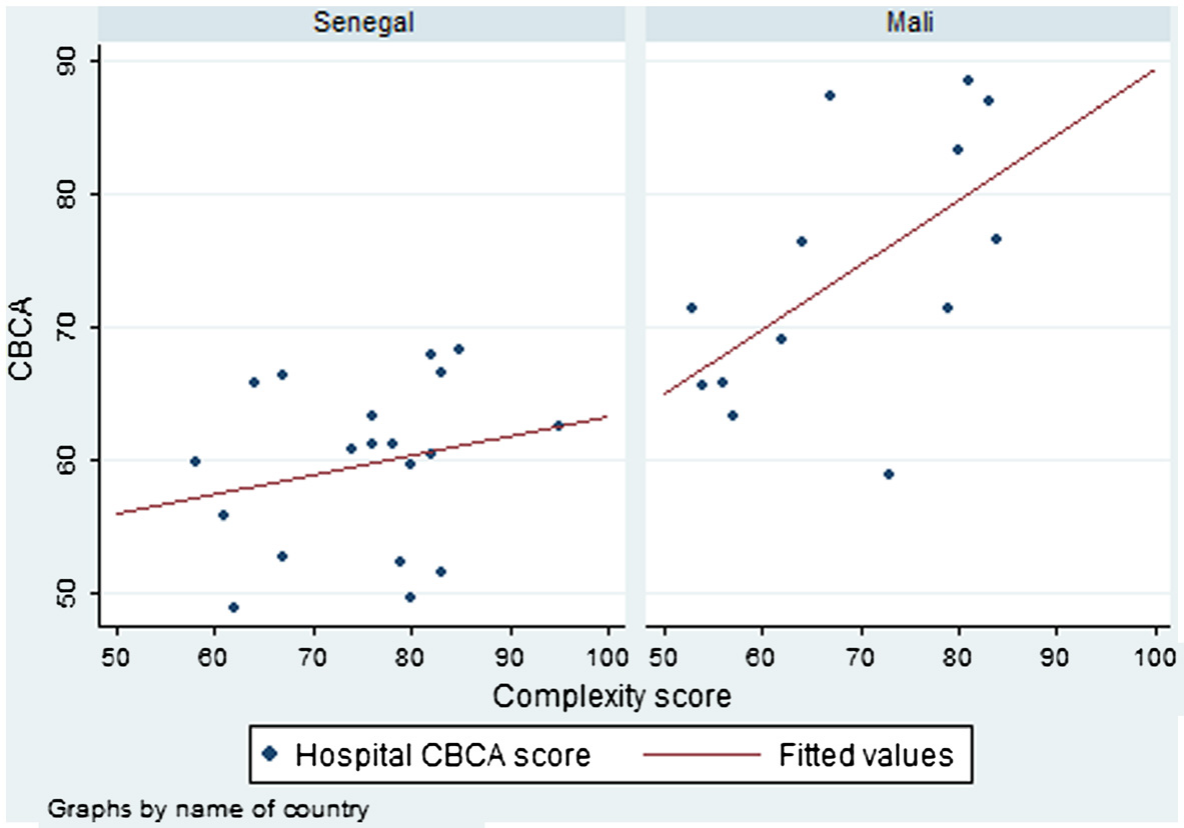
**Facility CBCA scores by facility complexity score according to country**.

Figure 2 shows the relationship between the Complexity Index and CBCA score by country. As hypothesized, there is a positive correlation between the two scores. This relationship is significant in Mali (Spearman’s Rho = 0.632, p = 0.010), but not in Senegal (Rho=0.293, p=0.112). In Senegal, there are three hospitals that appear to be outperforming, in terms of CBCA scores, hospitals with similar or greater Complexity Index scores. All three are rural, district-level hospitals (e.g. lowest level of referral hospital). When the three outliers are removed from the analysis, there is a significant linear relationship between Complexity Index and CBCA scores in Senegal (Spearman’s rho = 0.438, p= 0.045).

#### Correlation between hospital CBCA scores and hospital perinatal mortality

**Figure 3:**
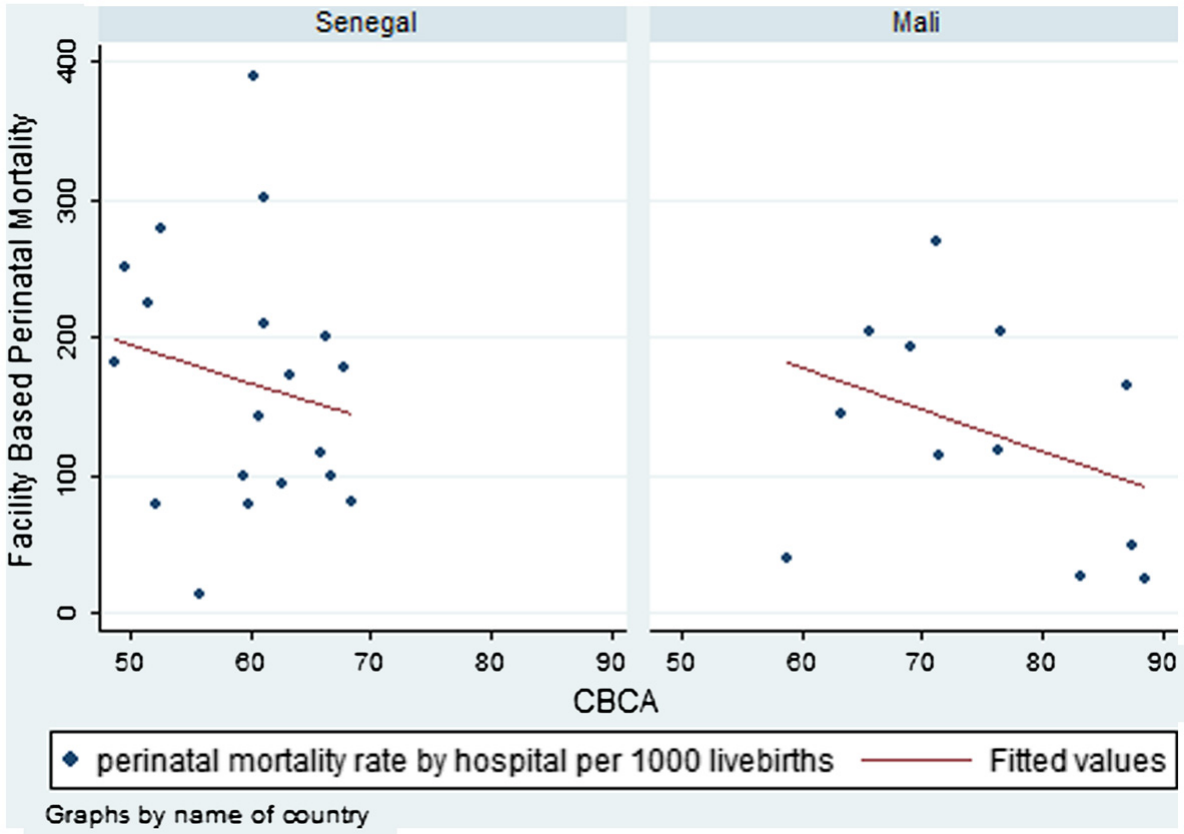
**Facility-based perinatal mortality by facility CBCA score according to country**.

Perinatal mortality was high in both countries. There were negative correlations between hospital CBCA scores and perinatal mortality (Figure 3). In Mali, the correlation coefficient was −0.35 (p=0.12); in Senegal, it was −0.18 (p=0.23). Because case mix is an important confounder between CBCA score and mortality outcome, we redid the analysis with only hospitals with at least 10% of patients with obstetrical complications. We thus removed 3 hospitals that were receiving and treating a population more akin to that of a community health centre than a referral site. Removing these sites, in Mali, the correlation coefficient was −0.57 (p=0.03) and in Senegal, it was −0.37 (p=0.07).

#### Correlation between hospital CBCA scores and hospital maternal mortality

**Figure 4:**
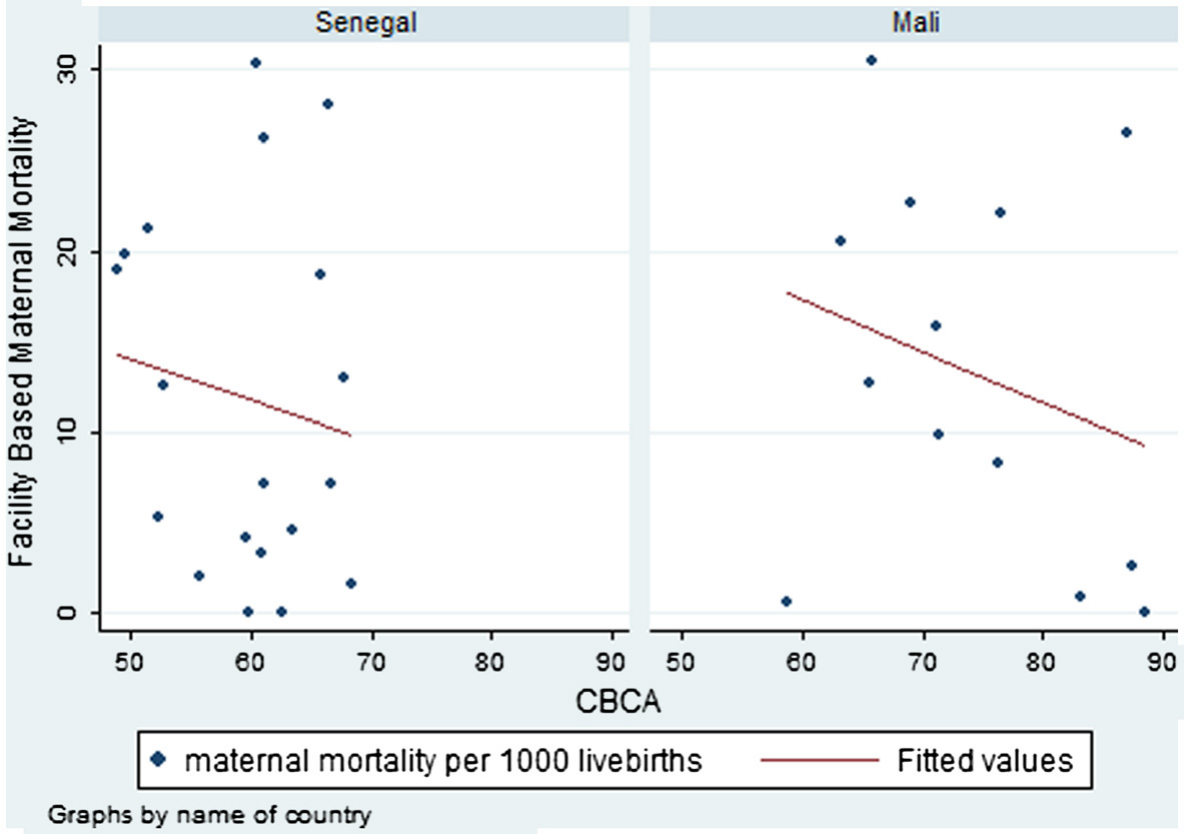
**Facility-based maternal mortality by facility CBCA score according to country**.

In both countries there were negative correlations between the hospital CBCA scores and facility maternal mortality (Figure 4). In Mali, the correlation coefficient was −0.25 (p=0.20) while in Senegal, it was −0.14 (p=0.28). As with the perinatal mortality analysis, we removed the three sites with very low levels of complication. With the restriction, the correlation between hospital CBCA scores and facility maternal mortality was −0.51 (p=0.045) in Mali and −0.25 (p=0.17) in Senegal.

#### Association between patient CBCA scores and perinatal outcomes

For this analysis, we merged the QUARITE and CBCA databases, pairing on the mother’s hospital registration number. In Mali, we had a 94.2% merging success and in Senegal, we had 91.9% merging success. In Mali, there were 44 (15.3%) cases of stillbirth and early neonatal death. In Senegal, there were 48 (14.3%) adverse perinatal outcomes but we could not calculate CBCA scores for two because of missing information in the medical charts. There were four cases of maternal deaths; all four had stillbirths.

Table 2 shows the number and percentage of stillbirths and early neonatal deaths for each section of the questionnaire according to those with good quality of care (> 70% criterion attainment) versus moderate to poor care (≤ 70% criterion attainment). It also shows the total number of women with criterion attainment above 70% for each questionnaire section. In Mali, for all sections of the questionnaire, there was a smaller proportion of stillbirths and early neonatal mortality in those patients with greater than 70% criterion attainment. In Senegal, the same is true for the first three sections of the questionnaire. Also, in Senegal, there were substantially fewer women (N=51) with greater than 70% criterion attainment than in Mali (N=171).

**Table 2:** Number and percentage of stillbirths and early neonatal deaths according to good (≥70% attainment) and moderate/poor (<70% attainment) quality of care

	**Mali**					**Senegal**				
	**Records indicating good quality of care (≥70%)**		**Records indicating poor quality of care (<70%)**		**p**	**Records indicating good quality of care (≥70%)**		**Records indicating poor quality of care (<70%)**		**p**
	**N**	**% stillbirths & neonatal deaths**	**N**	**% stillbirths & neonatal deaths**		**N**	**% stillbirths & neonatal deaths**	**N**	**% stillbirths & neonatal deaths**	
Patient History	273	14.7	14	28.6%	0.24	284	13.7	34	20.6	0.30
1^st^ Clin exam	277	15.2	10	20.0%	0.65	245	12.2	89	20.2	0.08
Laboratory	49	6.5	238	17.2%	0.05	93	7.5	238	17.2	0.02
Delivery	220	13.2	67	22.4%	0.08	56	19.6	278	13.3	0.22
Post-partum	74	10.8	213	16.9%	0.26	89	16.9	243	13.6	0.48
Total	171	11.1	116	21.6%	0.02	51	9.8	259	15.8	0.39

We did sensitivity analyses looking at different cut-offs for good quality of care (60, 70, and 80%). Our results suggest that the choice of 70% is adequate. In both countries, there was a smaller proportion of perinatal deaths in women with CBCA scores above 70% compared to 60%. No important change occurs after that cut-off, particularly in Senegal where very few women had CBCA scores above 75%.

In Table 3 we present the results of the generalized estimating equations adjusting for hospital and patient characteristics. In this analysis, age, parity, and number of prenatal visits were not confounders and we did not adjust for them. In all analyses, moderate to poor quality of care was significantly associated with stillbirth and neonatal mortality. Women with CBCA scores below 70% were approximately two times more likely to have a stillbirth or neonatal death (adjusted OR 1.84, p=0.05).

**Table 3:** Associations between CBCA score and stillbirth/early neonatal mortality

	**Crude odds ratios**		**Adjusted odds ratios**^**¥**^	
	**OR, P-value**	**95% CI**	**OR, P-value**	**95% CI**
**CBCA score**				
Good ≥70%	-	-	-	-
Poor <70%	1.85, p= 0.04	1.03-3.34	1.84, p=0.05	1.01-3.34
**Country**		-		
Mali	-		-	-
Senegal	0.89, p=0.68	0.51-1.56	0.59, p=0.11	0.31-1.12
**Location**				
Capital	-	-	-	-
Region	2.54, p=0.00	1.55-4.18	2.67, p=0.00	1.54-4.62
**Complexity index**	1.00, p=0.76	0.97-1.02	1.01, p=0.66	0.98-1.04
**Maternal condition**				
No	-	-	-	-
Yes	7.19, p=0.000	4.91-10.52	8.00, p=0.00	5.16-12.40

^¥^Adjusted for CBCA score, country, location, complexity index, and maternal condition.

## Discussion

To our knowledge, this is the first study to comprehensively assess the measurement properties of obstetrical CBCA. Our CBCA has elevated inter-rater reliability and there is compelling evidence of its validity; the tool performs according to theory at both hospital and patient levels of analysis. Average hospital CBCA scores positively correlate with Complexity Index scores and negatively correlate with hospital maternal and perinatal mortality. At the patient level, women with moderate to poor care had about 2 times the odds of perinatal death compared to women with good quality obstetrical care.

There are a number of strengths to this study. CBCA development was comprehensive and involved multiple revisions with international experts. Data collection was conducted by trained auditors using an electronic CBCA questionnaire with internal validity checks in order to reduce random error related to issues such as lost questionnaires and illegible writing. It is also one of the largest and most detailed audits conducted in a resource-limited setting. Finally, given concerns that obstetrical records could be missing in a non-random fashion, we systematically recorded the numbers of missing records and patient characteristics associated with missing obstetrical records. In Mali, we were able to retrieve 82.0% and in Senegal, we were able to retrieve 85.4% of obstetrical records. For both countries, there was no association between the patient age or type of birth (vaginal versus surgical) and the retrievability of the obstetrical record. This suggests that records were missing at random, as patient characteristics for retrieved and non-retrieved records were similar.

While this was a very large audit, there were sample size limitations related to the number of hospitals included (n=32). Estimates for the hospital-level analyses are unstable especially as considerable inter-country differences necessitated stratified analyses and thus reduced study power. In Mali, we observed significant associations while in Senegal, we mostly observed trends. Further, the hospital-level analyses did not adjust for confounding, such as differing levels of obstetrical complication. In theory, all hospitals are referral sites and should treat relatively similar proportions of complicated deliveries. However, certain hospitals appeared to receive a disproportionately low number of complications (less than 10%). These sites have serious organizational dysfunctions that place in question their categorization as comprehensive obstetrical referral sites. When we excluded these outlying hospitals, all correlations intensified.

In this paper, our definitions for perinatal and maternal mortality reflected the fact that this was a hospital-based study and the goal of the larger QUARITE trial was to reduce facility mortality rates. Thus, we only followed women and their newborns until discharge. We did not include cases of perinatal and maternal deaths that occurred after leaving the hospital. In the case of perinatal mortality, the fact that we could not follow newborns for a full week after birth entailed that our sample size for perinatal mortality was lower than expected because of few recorded early neonatal deaths (n=11). Based on previous studies [25], we expected approximately equivalent numbers of stillbirths and early neonatal deaths. We believe that a lack of postpartum monitoring (on average, less than 50% of criteria for this section were attained) meant that cases of early neonatal mortality were not detected and/or recorded. Despite this limitation, we observed that perinatal mortality was between 1.5 and 2.0 times higher in women with lower than 70% criterion attainment. This result is consistent with the odds ratio obtained by the generalized estimating equations which adjusted for both hospital and patient characteristics and also accounted for the clustering effect of the study design.

Because of the small number of neonatal deaths, our study outcome for the patient analyses consisted mostly of stillbirths. One weakness of our study is that we did not remove cases of intrauterine death from this outcome. We did not do so because information on intrauterine death was inconsistently recorded by the study. In Senegal, the foetal heart rate was not evaluated in 16% of women while this was only the case for 2% of women in Mali. By not removing cases of probable intrauterine death, we likely introduced non-differential misclassification which typically biases the estimate towards the null.

The most important weakness of using CBCA is that the instrument is dependent on what is recorded in patient medical charts. If this information is incomplete or inaccurate, it can introduce measurement error. For example, during the audit, we could not find the vital status of 23 infants at birth. We were fortunate to have a second database to recuperate missing data but, nevertheless, made the assumption that four cases of recorded livebirths were in fact perinatal deaths given their very low birth weights. This assumption was also based on CBCA results indicating that these four women had no postpartum monitoring. To assure that we had not introduced error, we did two additional analyses treating these four cases as livebirths and as missing data; in all analyses, we obtain adjusted point estimates for CBCA score within two tenths of each other. Overall, we noted that the quality of data recording was generally poorer in Senegal compared to Mali, as evidenced by the lower reliability score and the non-overlapping confidence intervals. Reliability and validity coefficients are interlinked. The reliability of a test puts a cap on the possible validity for that test. Poorer data recording in Senegal introduced random error that reduced the precision of the CBCA instrument.

## Conclusion

Overall, in conjunction with the elevated reliability coefficients, we feel that the converging evidence from the multiple assessments of construct validity in this article provide compelling evidence of the utility of this instrument to measure intrapartum quality of care. CBCA has the advantage of measuring the actual obstetrical practice received by a patient (compared to provider knowledge with vignettes) and is less subjective than expert observation. It was originally developed as a quality improvement tool, but has promising research applications and can thus benefit both researchers and clinicians in measuring and improving obstetrical quality of care. As we have previously argued [15], recommendations based on clinical audits need to be based on valid and reliable instruments. This tool helps fill that gap.

## Abbreviations

CBCA: Criterion-based clinical audit.

## Competing interests

The authors declare that they have no competing interests.

## Authors’ contributions

All authors were involved in study conception. CP and AD participated in all steps of the research study. MT was instrumental in questionnaire development and results interpretation. MVZ contributed to the statistical analyses, results interpretation, article writing, and revision. All authors read and approved the final manuscript.

## Authors' information

CP is a PhD student in public health and epidemiology at the Université de Montreal. She has spent the last 6 years working in West Africa on maternal and child health and HIV/AIDS. She is a Vanier Canada Graduate Scholar. AD is obstetrician gynaecologist and public health researcher. He has spent the past 15 years working in maternal health in West Africa, especially Senegal. MT is professor of gynaecology at the Université de Bamako, Mali, and has worked intimately with the QUARITE research team since project inception (2007). MVZ is a professor of epidemiology at the Université de Montréal and has over 20 years of experience in global health research. She has done extensive work on questionnaire development and validation.

## Ethics approval

The QUARITE trial is registered on the Current Controlled Trials website under ISRCTN46950658. The trial received ethics committee approval from Ste Justine Hospital in Montréal (ref. 2425), the Ministry of Health and Preventive Medicine in Senegal (ref. 0869), and the National Ethics Committee for Health and Life Sciences in Mali (ref 034/MS-SG-CNESS). Informed consent was obtained from each hospital included in the trial.

## Pre-publication history

The pre-publication history for this paper can be accessed here:

http://www.biomedcentral.com/1471-2393/12/118/prepub

## Supplementary Material

Additional file 1: **Supplementary materials (English Translation- CBCA Instrument).** (DOC 35 kb) Click here for file
