# Supplementary material for: Validity and reliability of criterion based clinical audit to assess obstetrical quality of care in West Africa
Source: BMC Pregnancy Childbirth. 2012 Oct 29;12:118. doi: 10.1186/1471-2393-12-118 (PMC3514290; doi:10.1186/1471-2393-12-118)
Supplement: Additional file 1: — Supplementary materials (English Translation- CBCA Instrument). (DOC 35 kb) [file 1471-2393-12-118-S1.doc]

CBCA INSTRUMENT

Identification

Complete the sections below.

Country________________________________________________

Health Structure__________________________________________

Registration Number_____________­­­­_________________________

Patient admission date_____________________________________

Time of admission________________________________________

Patient initials___________________________________________

For all women- patient history

Select one of the options below. If the information is not available in the patient record, select not provided.

1) Condition of the mother at admission

□ Not provided

□ Good physiological condition

□ Medium physiological condition

□ Critically-ill

□ Very critically-ill

Check yes if the information is available in the medical record and no if it is unavailable. If it is available, provide the corresponding information (e.g. the number of recorded prenatal exams, etc).

2) Number prenatal visits □YES________________□NO

3) Age □YES________________□NO

4) Gravidity □YES________________□NO

5) Parity □YES________________□NO

COntinue to next page

For all women- first clinical examination

These questions pertain to the first clinical examination of the patient. Check yes if the information is available in the medical record and no if it is unavailable. If it is available, provide the corresponding information (e.g. measure of uterine height, etc.).

6) Uterine height □YES________________□NO

7) Cardiac frequency (pulse) □YES________________□NO

8) Blood pressure □YES________________□NO

9) Temperature □YES________________□NO

10) Fœtal presentation □YES________________□NO

These questions pertain to the first clinical examination of the patient. Check yes if the information is available in the medical record and no if it is unavailable.

11) Fœtal heart beat □YES □NO

12) Integrity of the membranes and/or colour of the amniotic fluid □YES □NO

13) Measure of cervical dialation □YES □NO

For all women- Laboratory Examinations

Check yes if the laboratory result is available and recorded in the medical record and no if this information is not available.

14) Blood type □YES □NO

15) Rhesus factor □YES □NO

16) HIV test □YES □NO

17) Syphilis test □YES □NO

FOR ALL WOMEN- DELIVERY AND BIRTH

These questions apply to the time of delivery and birth. Check yes if the information is available in the medical record and no if it is not available.

18) Name of the birth attendant □YES □NO

19) Time of the placental expulsion □YES □NO

**CONTINUE TO NEXT PAGE**

Select one of the options below. If the information is not available, select no.

20) Birth assisted by a skilled attendant

□No

□Yes- doctor

□Yes- midwife

□Yes- resident in obstetrics/gynaecology

□Yes- nurse

□Yes- birth assisted but qualification not recorded

21) Intramuscular or intravenous oxytocin administered to the woman during delivery or immediately post-partum?

□No

□Yes- as part of the active management of the third phase of labour (AMTSL)

□Yes- oxytocin only

22) Time of birth □Yes (*write in the time*) _____________ □No

For all women- Follow-up after birth

Select one of the options below.

23) Was there clinical follow-up of the woman after birth (including the height of the uterine fundus and at least one vital sign)?

□Yes, within the two hours following birth

□Yes, between two and four hours after birth

□Yes, four or more hours after birth

□No, follow-up not noted recorded

Check yes if the information is available in the medical record and no if the information is not available.

24) Was a medical examination conducted prior to the woman’s hospital discharge? □YES □NO

25) Was the woman’s date of discharge recorded? □YES □NO

**CONTINUE TO NEXT PAGE**

*Select one of the options below.*

26) Was the vital status or APGAR score of the new-born recorded in the medical record?

□No

□Yes

□N/A Stillborn

**END OF CBCA INSTRUMENT**
